# Ionic conductivity enhancement in solid polymer electrolytes by electrochemical *in situ* formation of an interpenetrating network†

**DOI:** 10.1039/d0ra07966a

**Published:** 2020-11-12

**Authors:** Kristian Leš, Carmen-Simona Jordan

**Affiliations:** University of Applied Sciences Osnabrück Albrechtstr. 30 49076 Osnabrück Germany k.les@hs-osnabrueck.de s.jordan@hs-osnabrueck.de

## Abstract

Various overoxidized poly(1*H*-pyrrole) (PPy), poly(*N*-methylpyrrole) (PMePy) or poly(3,4-ethylenedioxythiophene) (PEDOT) membranes incorporated into an acrylate-based solid polymer electrolyte matrix (SPE) were directly electrosynthesized by a two-step *in situ* procedure. The aim was to extend and improve fundamental properties of pure SPE materials. The polymer matrix is based on the cross-linking of glycerol propoxylate (1PO/OH) triacrylate (GPTA) with poly(ethylene glycol) diacrylate (PEGDA) and lithium bis(trifluoromethanesulfonyl)imide (LiTFSI) as a conducting salt. A self-standing and flexible polymer electrolyte film is formed during the UV-induced photopolymerization of the acrylate precursors, followed by an electrochemical polymerization of the conducting polymers to form a 3D-IPN. The electrical conductivity of the conducting polymer is destroyed by electrochemical overoxidation in order to convert the conducting polymer into an ion-exchange membrane by introduction of electron-rich groups onto polymer units. The resulting polymer films were characterized by scanning electron microscopy, cyclic voltammetry, electrochemical impedance spectroscopy, differential scanning calorimetry, thermal analysis and infrared spectroscopy. The results of this study show that the combination of a polyacrylate-matrix with ion selective properties of overoxidized CPs leads to new 3D materials with higher ionic conductivity than SPEs and separator or selective ion-exchange membrane properties with good stability by facile fabrication.

## Abstract 2

Conductive polymers were encapsulated and subsequently overoxidized in an acrylate polymer matrix as potential separator materials *via* the combination of UV-induced and electrochemical polymerization.

## Graphical abstract

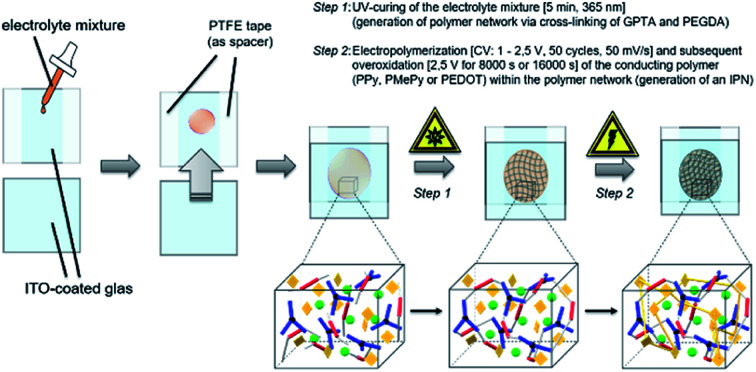

## 1. Introduction

The release of the lithium-ion battery (LIB) in 1991 by the Sony Corporation marked an important milestone towards the development of energy storage systems, especially rechargeable batteries.^1,2^ Vast progress has been made since then on the improvement of materials and properties that enabled the implementation of the LIB in different markets.^3,4^ Therefore, more and more products from different fields of technology tend to have nowadays LIBs as power sources, for example small portable electronic devices or plug-in electric vehicles (*e.g.* cars, scooters, buses), which gained an increase in popularity due to recent climate changes and government incentives.^5,6^ The call for environment-friendly power sources is more topical than ever.^7^ One major criteria is accordingly the safety of the integrated battery and its components.^8^ The conventional LIB with liquid electrolytes provides a high lithium ion conductivity, but suffers from safety issues that are related to the flammability of organic solvents and decomposition products.^9,10^ Hence, alternative electrolytes based on polymer matrices without organic solvents (SPEs) have been the research subject for many years now. The SPEs can be modified to prepare different kinds of polymer electrolyte variations, namely gel polymer electrolytes (GPE) or composite polymer electrolytes (CPE).^11^ SPEs have distinct advantages to liquid electrolytes that are related to mitigation of lithium dendrites and slower electrode/electrolyte interfacial deformation, as well as thermal, mechanical and electrochemical stability.^9^ While fulfilling the safety criteria, the ionic conductivity of such polymer electrolytes is generally lower than in liquid electrolytes. First studies on the electrical conductivity of the ionic polymer polyethylene oxide (PEO) were published in 1975 by Wright.^12^ That discovery led to the application of PEO with mixed lithium salts as SPE in lithium secondary batteries by Armand *et al.*^13^ The ionic conductivity of such polyether–lithium salt complexes ranges in the order of 10^−8^ to 10^−7^ S cm^−1^ at room temperature (RT), because of the high crystallinity at ambient temperatures.^11,14^ The ionic transport in SPE takes normally place in an amorphous state throughout segmental motion of the host polymer.^15^ Thus, various approaches were developed to lower the glass transition temperature (*T*_g_) and improve the overall ionic conductivity of SPEs.^9,16–19^ Modifications include polymer/polymer coordinating electrolytes with cross-linked polymers, composite polymers, block/comb copolymers, interpenetrating network polymers and blend polymers.^20,21^

A particularly interesting approach to make use of different advantages is the preparation of composite polymer electrolytes with interpenetrating polymer networks (IPN). IPNs combine two or more cross-linked polymers without any covalent bonds between these individual polymers. Based on different researches, IPNs have the capability to decrease crystallinity and therefore enhance ion conductivities and mechanical properties.^21^ Zeng *et al.* prepared a solvent-free poly(ether-acrylate) IPN with high conductivity at RT (2.2 × 10^−4^ S cm^−1^), low glass transition temperature (−64.2 °C) and a major effect on the blocking of Li dendrite growth.^22^ Another variation by Duan *et al.* was the preparation of an *in situ* plasticized double-network SPE with similar good results in ion conductivity (from 10^−5^ to 10^−4.5^ S cm^−1^ at RT), electrochemical stability (4.7 V *vs.* Li/Li^+^), thermal stability (up to 200 °C) and Li dendrite suppression.^18^

Apart from lithium-ion battery applications, IPNs are overall popular systems in other energy-related research fields, *e.g.* for supercapacitors,^23^ solar cells^24^ or actuators.^25^ Fong *et al.* devised a supercapacitor electrode comprising an electronically conducting, pseudocapacitive polymer (PEDOT) within an ionically conducting polymer matrix. The framework of this semi-IPN offers the possibility to develop new interpenetrating materials based on conducting polymers (CPs).^23^ Some researchers transferred the idea of conducting polymers to LIBs, mostly as materials for high-performance electrodes and its components or as separator composites.^26–31^ When applied as separator, the electronic nature of CPs should be eliminated to advert the risk of a short circuit between the positive and negative electrode of a LIB. Wang *et al.* presented two different methods to introduce CPs as separator materials: an overoxidized (base and heat-treatment process) polypyrrole/cellulose composite as separator with good thermal stability and high electrolyte wettability, as well as a bilayered redox-active separator composed of insulating nanocellulose fibers and capacity-enhancing polypyrrole.^30,31^ Both separator variations were developed as replacements for conventional separators in LIBs, which contain organic liquid electrolytes. An approach by J. Amanokura *et al.* was the fabrication of a polypyrrole/polymer electrolyte (PE) composite as cathode/electrolyte material. The preparation included an *in situ* electropolymerization of pyrrole in a PE matrix. According to the achieved theoretical redox potential of PPy *vs.* Li/Li^+^ and a charge–discharge performance of the PPy/PE/Li cells, the researchers suggested a potential application in solid-state electrochemical cells.^32^ Nevertheless, none of the currently known publications focuses on the implementation of overoxidized CPs, such as oPPy, into polymer matrices as separator materials for LIBs. Overoxidation of PPy at high potentials has been reported by many researchers.^30,31^ In contrast to reversible oxidation of PPy, overoxidation is irreversible, which causes a loss of conductivity and formation of carbonyl groups that can attract cations and hinder the diffusion of anions through the film. Based on this, overoxidation offers an additional advantage regarding the properties and applications of conducting polymers. Overoxidized CPs are widely applied in the field of electrochemical sensing, biosensing, ion exclusion or permselective membranes *etc.*^23,30–32^

In this work, we propose a new two-step “*in situ*” procedure to prepare an interpenetrating solid polymer electrolyte (I-SPE) based on cross-linked acrylates and electrochemically overoxidized conducting polymers. The aim is to extend and improve fundamental properties of pure SPE materials. The first step being the photopolymerization of the acrylate matrix and the second one is the electrosynthesis of conductive polymer films from electroactive monomers embedded into the acrylate matrix and its overoxidation. The second step can last several hours, until the anodic current is very low and constant. The performances of I-SPE membranes were characterized in terms of electrochemical properties, ionic conductivity, thermal stability and morphology. Here, we combined the advantage of a polyacrylate-matrix with cation selective property of overoxidized PPy, PMePy and PEDOT by electropolymerization to construct new 3D structures with higher ionic conductivity than SPEs and separator or selective ion-exchange membrane properties with good stability by facile fabrication.

We have demonstrated that the use of different overoxidized conductive polymers incorporated into a polymer matrix provides an alternative way of varying the properties and applications of conducting polymers.

## 2. Experimental

### 2.1 Materials

Glycerol propoxylate (1PO/OH) triacrylate (GPTA, *M*_n_ ≈ 428 g mol^−1^), poly(ethylene glycol) diacrylate (PEGDA, *M*_n_ = 700 g mol^−1^), 2,2-dimethoxy-2-phenylacetophenone (DMPAP, 99%), lithium bis(trifluoromethanesulfonyl)imide (LiTFSI, ≥99%), *N*-methylpyrrole (99%) and EDOT (97%) were purchased from Sigma-Aldrich. 1*H*-Pyrrole (99%, extra pure) was bought from ACROS and freshly destilled prior to use. All chemicals were stored and processed under argon atmosphere in a glove box.

### 2.2 Preparation of the solid polymer electrolyte (SPE)

The SPE film was prepared by stirring GPTA (18 wt%), PEGDA (72 wt%) and LiTFSI (10 wt%) together. In terms of raising the ionic conductivity of the matrix, the most efficient weight-based ratio of GPTA : PEGDA was found to be 1 : 4. The radical photoinitiator DMPAP was added afterwise to the solution in a proportion of 2 wt% relative to the GPTA/PEGDA content. Once all components were homogeneously dissolved, the precursor solution was spread and polymerized between the flat-bottomed sides of two Petri dishes. The photopolymerization was carried out by UV-irradiation for 5 min using a UV lamp (8 W, 365 nm, model MLR-58, UVP Ultra-Violet Products Ltd.). The SPE was obtained as a flexible and robust free-standing polymer film with a thickness of 150 μm.

### 2.3 Preparation of the interpenetrating solid polymer electrolyte (I-SPE)

**Fig. 1:**
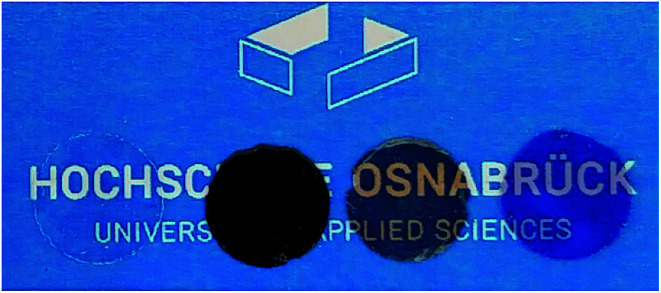
Picture showing the prepared SPE, I-SPE-oPPy, I-SPE-oPMePy and I-SPE-oPEDOT (from left to right) as round discs with a diameter of 11 mm.

**Fig. 2:**
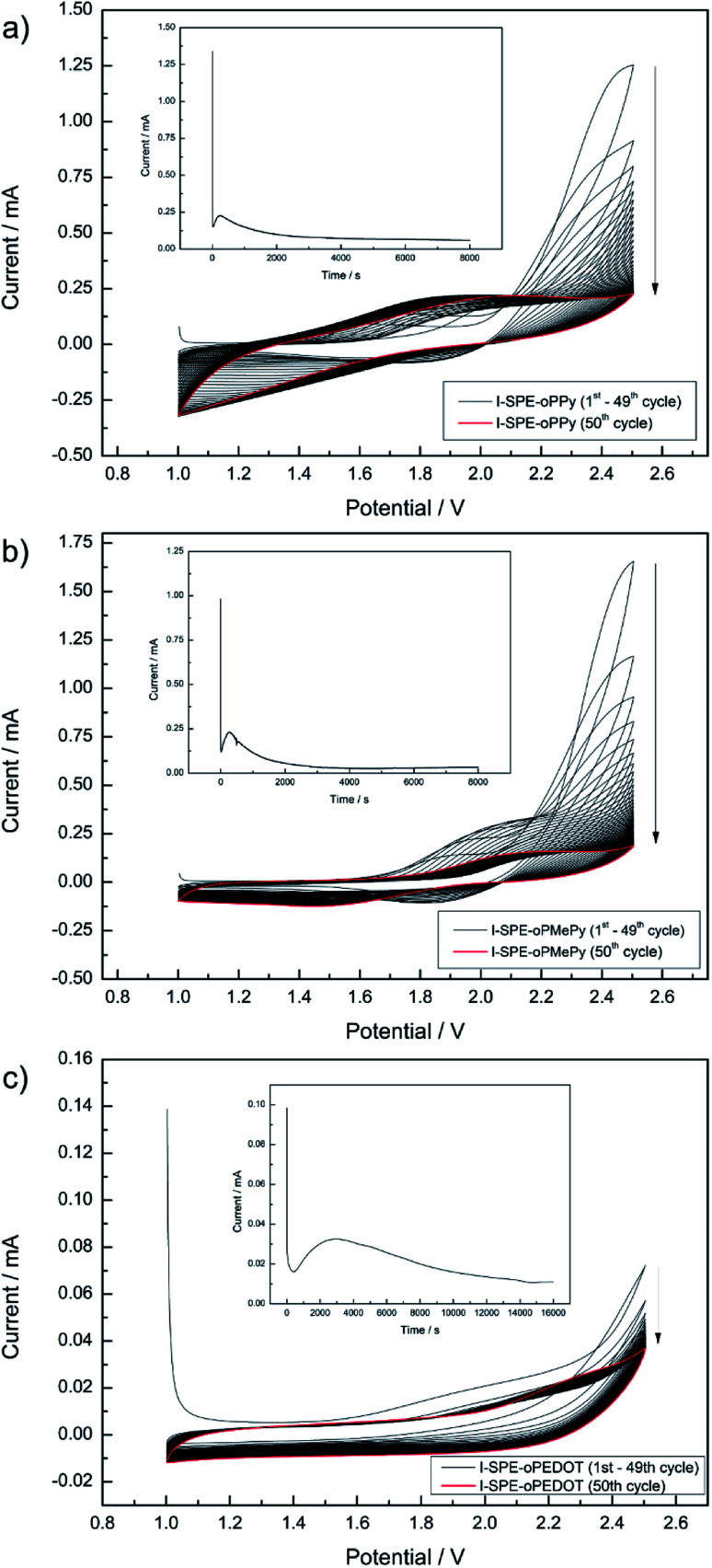
Cyclic voltammograms and chronoamperograms (as insets) of the electropolymerization procedure for I-SPE-oPPy (a), I-SPE-oPMePy (b) and I-SPE-oPEDOT (c).

**Scheme 1:**
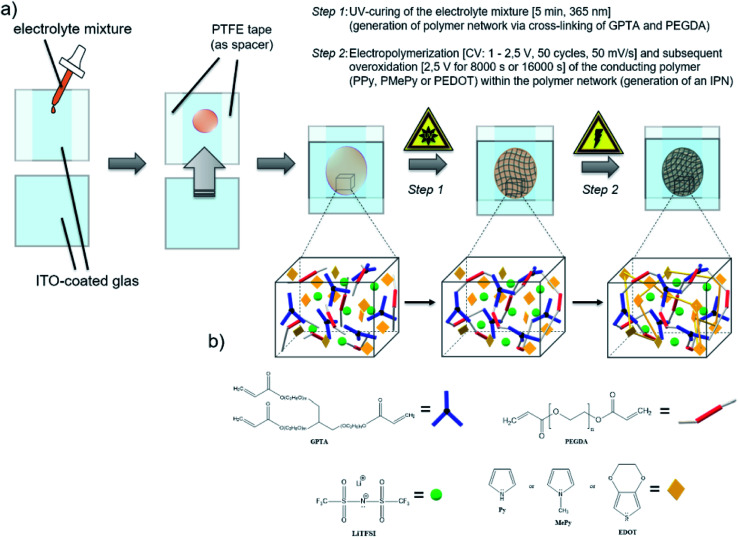
Schematic illustration of (a) the practical preparation of the I-SPE by photochemical and electrochemical polymerization, (b) the interpenetrating polymer network build-up by acrylic and cyclic monomers.

To the precursor solution of GPTA, PEGDA and LiTFSI either PPy, MePy or EDOT monomer was added, so that the weight ratios became 14/56/10/20, respectively. The mixture was stirred thoroughly. After the photoinitiator (2 wt% of the acrylate monomers) was added, the mixture was spread between two indium-tin oxide (ITO) glass plates (50 × 50 mm, 1.1 mm thick, 1850 Å ITO layer on one side, resistivity of 10–15 Ω per square inch, Adafruit Industries). To avoid contact of the ITO plates, a PTFE thread sealing tape (12 mm × 0.1 mm × 12 m, 60 g m^−2^) was wrapped as spacer around the edges of one ITO plate. In the first step of the polymerization, the cross-linking of the GPTA/PEGDA network was performed by UV-irradiation for 5 min. After that, the electrochemical polymerization of the conducting polymer component was carried out *in situ* by contacting the ITO plates as working and counter electrode to a potentiostat (Metrohm Autolab PGSTAT302N). A scheme of the procedure is illustrated in Scheme 1. First, cyclic voltammetry was performed for 50 cycles in a potential range from 1 V to 2.5 V at a scan rate of 50 mV s^−1^. The number of cycles depends on the amount of the conducting polymer in the formulation and its complete polymerization. After that, a constant potential of 2.5 V was applied for 8000 s until no more current response was detected, indicating the loss of electroactivity. The chronoamperometric procedure for I-SPE-oPEDOT was performed for 16 000 s. The associated cyclic voltammograms and chronoamperograms are depicted in Fig. 2. After the procedure, the ITO plates were detached from each other and a coloured, less-transparent and free-standing I-SPE membrane was obtained with a thickness of about 100–140 μm (Fig. 1).

### 2.4 Characterization methods

The determination of the ionic conductivity (*σ*) of the electrolyte membranes was carried out *via* electrochemical impedance spectroscopy (EIS) on an Autolab potentiostat. The electrolyte membranes were cut into round disks by using a hollow punch (Forum®, 11 mm). The thickness of the membranes was measured using a micrometer screw gauge (Helios, 0–25 mm, 0.01 mm). EIS was performed in a symmetrical electrode setup by sandwiching the membrane between two stainless steel electrodes (SS/SPE/SS), whereas the ionic conductivity was calculated using following equation, where *l* is the thickness, *R*_b_ is the bulk resistance and *A* is the area of the probe:1
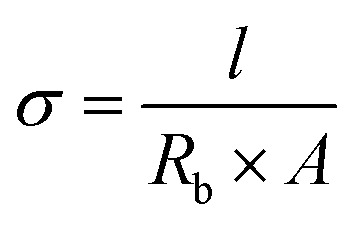


The measurement was performed within a frequency range of 10^6^ Hz (or 10^5^ Hz) to 1 Hz with an AC amplitude of 20 mV at RT. Regarding the electrochemical performance, all measurements were carried out in a two-electrode cell with glass housing and stainless steel parts. Temperature-dependent measurements were carried out in an oven (UM100, Memmert GmbH) ranging from 30 to 80 °C. The activation energy of lithium ion conduction was calculated by linear fitting of the Arrhenius plots in accordance with following Arrhenius equation, where *σ*_0_ is the pre-exponential factor, *E*_a_ is the activation energy and *k* is the Boltzmann constant:2
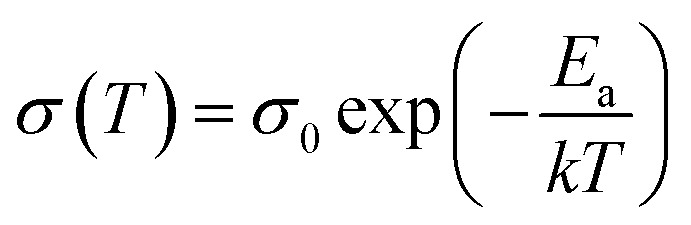


Thermal properties of the polymer membranes were characterized by thermogravimetric analysis (TGA, TG 209 F1 Libra®, Netzsch) in a temperature range from 28 to 600 °C under argon atmosphere and at a heating rate of 5 °C min^−1^. Differential scanning calorimetry (DSC 3500 Sirius, Netzsch) was carried out at a heating/cooling rate of 5 °C min^−1^ under argon atmosphere. Two cycles were performed in the range from −50 to 150 °C. The surface morphology of the samples was examined with a focused ion beam scanning electron microscope (FIB-SEM, FEI Helios NanoLab 600). For this purpose, the electrolyte membranes were sputtered with a thin layer of PtPd (approx. 3 nm). The imaging was conducted with an accelerating voltage of 10 kV and at a magnitude of 10 000 times. Attenuated total reflectance (ATR-FT-IR, Platinum-ATR, Bruker) was performed to evaluate the conversion characteristics in the SPE and I-SPE.

## 3. Results and discussion

### 3.1 Structural properties

**Fig. 3:**
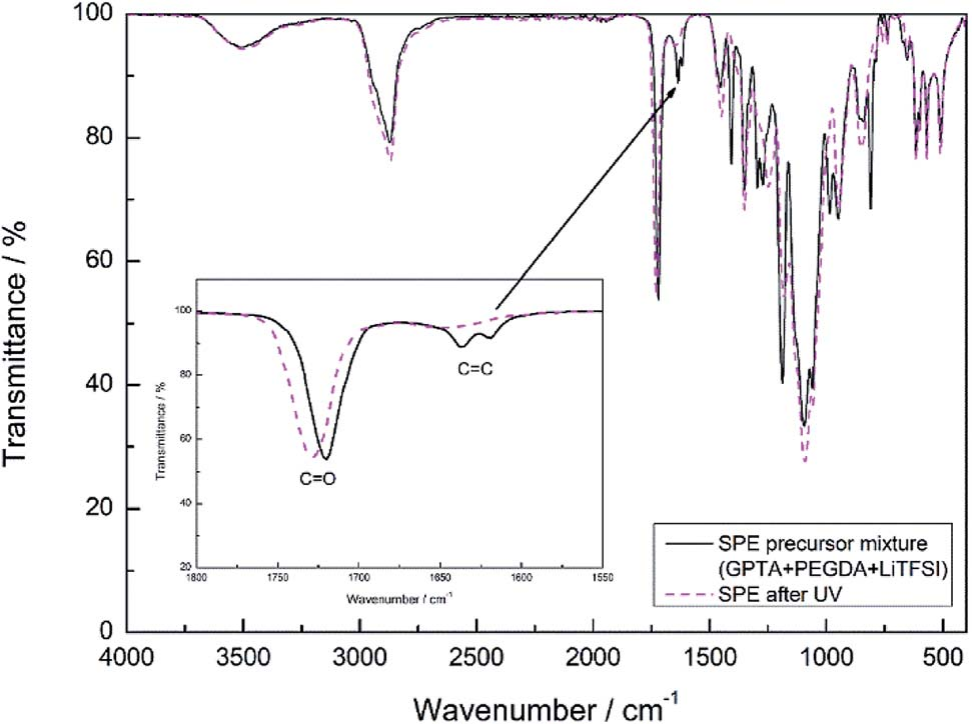
ATR-FT-IR spectra of the SPE formulation before and after UV-irradiation.

**Fig. 4:**
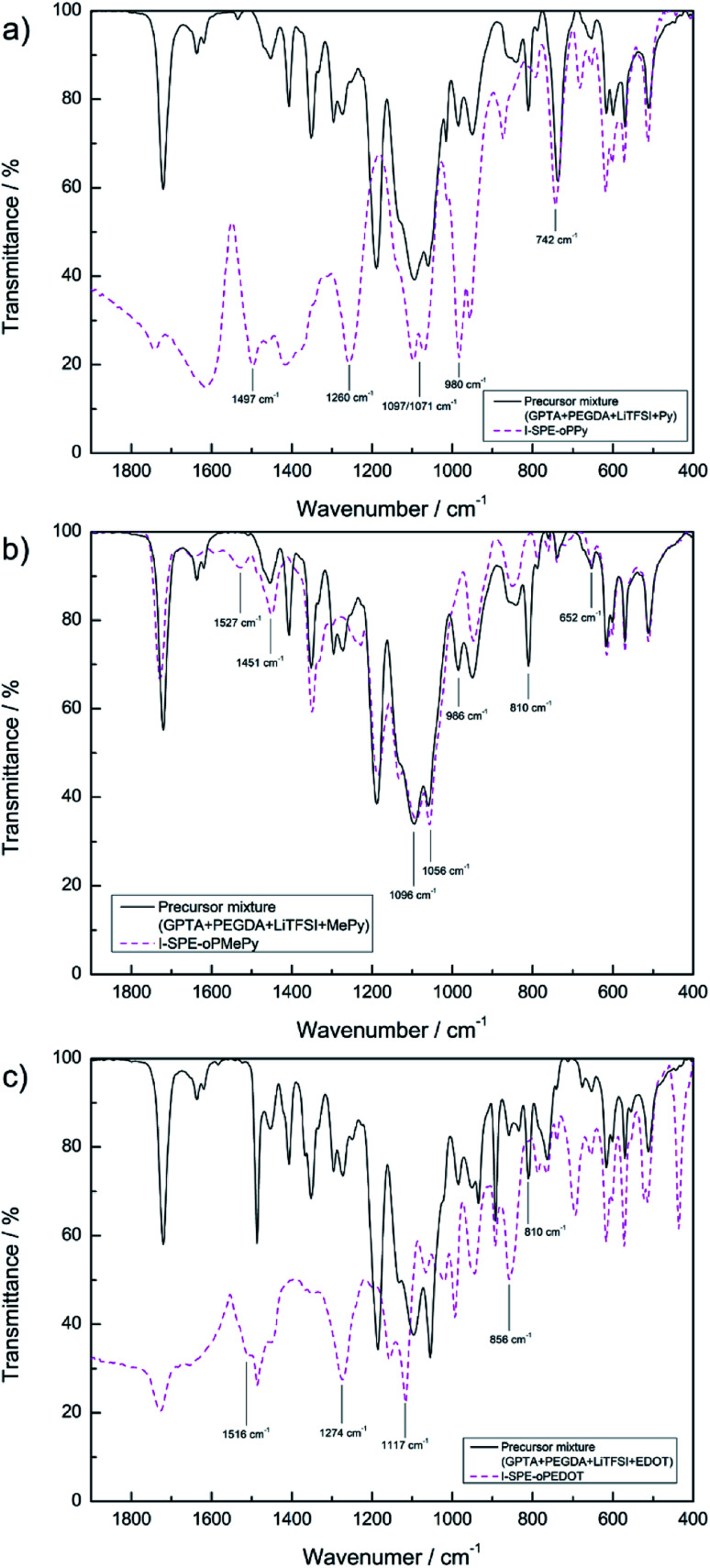
ATR-FT-IR spectra of (a) I-SPE-oPPy, (b) I-SPE-oPMePy and (c) I-SPE-oPEDOT as precursor mixtures and after UV-irradiation and ECP procedure.

The polymerization process of the SPE and I-SPE was examined *via* ATR-FT-IR spectroscopy (400–4000 cm^−1^). Fig. 3 shows the absorbance FT-IR spectra of the SPE before and after the UV-irradiation. The characteristic peak of the acrylic group (CH_2_=CH–COR) at 1600–1650 cm^−1^ disappears after the photopolymerization, implying a successful cross-linking of the GPTA and PEGDA acrylate groups.^33^ FT-IR spectra of I-SPE-oPPy, I-SPE-oPMePy and I-SPE-oPEDOT were carried out as precursor mixture and after the UV- and electropolymerization process. The results are shown in Fig. 4a–c. Typical peaks give information on the formation of the conjugated polymer and its overoxidation, which is encapsuled in the acrylate matrix. The interpretation of such composites, in particular of polypyrrole, is still challenging, because of the variety in preparation methods, doping levels, conjugation lengths, broadening of absorption bands and the overlapping of vibration modes from different molecules in the sample.^34,35^

Fig. 4a shows the absorption curve of I-SPE-oPPy. The high frequency region (>2000 cm^−1^) has been omitted, as it lacks defined peaks. The distinct peaks at 1497, 1260, 980 and 742 cm^−1^ represent the C–N stretch vibration, the N–H/C–H in-plane bending, the ring deformation and the out-of-plane vibration of polypyrrole, respectively.^36–39^ The double peak at 1071/1097 cm^−1^ could be the C–O–C stretch vibration of the PEG unit in PEGDA,^40^ since it is also present in the SPE spectra before and after UV-polymerization. The conjugation of polypyrrole is usually expressed as characteristic peaks at about 1480 and 1560 cm^−1^, which describe the symmetric and anti-symmetric ring vibration.^41^ A shoulder peak at 1460 cm^−1^ is recognizable in the spectrum, but there is no peak at around 1560 cm^−1^. This could be an indication of overoxidation. It has been reported that the overoxidation of polypyrrole leads to reactions, which decrease the conjugation length, form the C=O functional group in the polymer backbone or additionally show ring-openings.^31,42,43^

The absorption curve of I-SPE-oPMePy (Fig. 4b) shows similar characteristics as the polypyrrole composite, because of the pyrrole backbone. Following fundamental peaks can be assigned to PMePy: 1096 cm^−1^ (C–H deformation) and 1056 cm^−1^ (C–H out-of-plane stretching).^44–46^ As it has been reported previously, the *N*-substituted methyl group (C–H stretching) can show absorption around 1350–1450 cm^−1^ and 663 cm^−1^.^46–48^ From the spectrum, the peaks at 1451 (CH_3_ asymmetric deformation) and 652 cm^−1^ (ring deformation with N–CH_3_ stretching) are in agreement to the reported values. The disappearing of two peaks after the polymerization process indicates the overoxidation of the polymer. The peaks at 810 and 986 cm^−1^ of the pre-polymerization mixture are not present in the curve after the polymerization. Both peaks reflect the C–H bending (out-of-plane and in-plane bending, respectively) in α-positions to the nitrogen atom. Furthermore, the peak at 1527 cm^−1^ (C=C stretching) is only present in the spectrum after the polymerization, which could be related to the α-coupling.

Fig. 4c shows the spectrum of I-SPE-oPEDOT before and after polymerization. The relevant shoulder peak at 1516 cm^−1^ (symmetrical C=C stretching of the thiophene ring) and the disappearance of the peak at 810 cm^−1^ (C–H bending) indicate successful polymerization. The vibration mode of the thiophene ring can be observed at 856 cm^−1^ (C–S stretching). The strong peaks at 1117 cm^−1^ and 1274 cm^−1^ may result from overoxidation, which can be assigned to symmetric and asymmetric vibrations from SO_2_.^49^ A sulfone group is usually formed upon overoxidation of polythiophene, but it is assumed that the additional ethylene-dioxy group in PEDOT does not change the fundamental overoxidation mechanism.^50^

### 3.2 Thermal properties

**Fig. 5:**
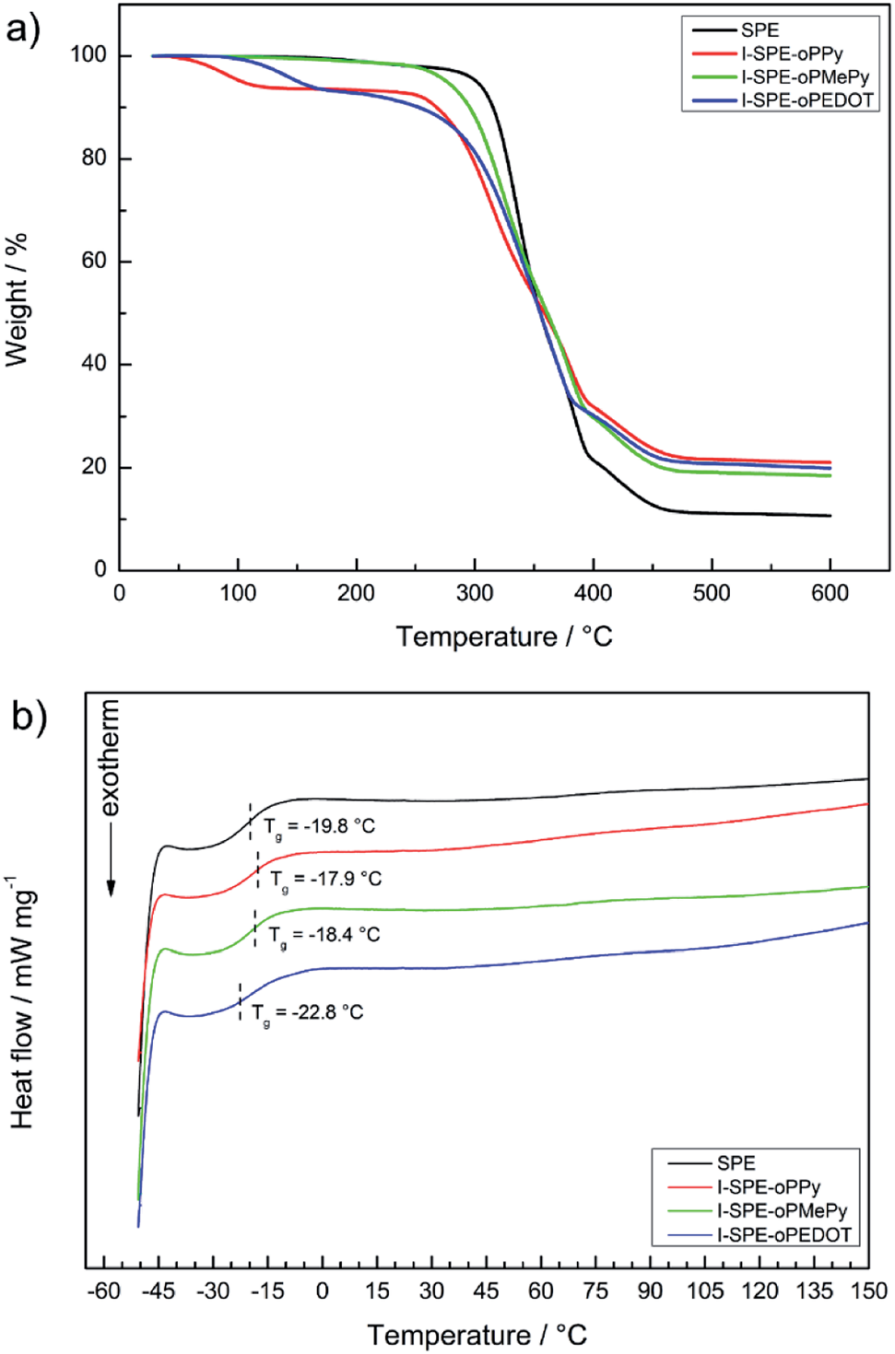
Thermal analysis of the SPE and its three IPN-modifications: (a) TGA thermograms, (b) DSC curves.

Thermogravimetric analysis and differential scanning calorimetry were performed to determine the temperature-dependent stability of the polymer matrix and the composite materials. It can be seen from the TGA thermogram in Fig. 5a, that the major weight loss processes result from the decomposition of the acrylate-based polymer network, the incorporated overoxidized polymers and the lithium salt. A small percentage of residual weight loss before the first major decomposition can be attributed to the hydroscopic nature of the materials and residual water evaporation consequently. It is known from literature reports that especially neat polypyrrole can show such weight loss before its intrinsic decomposition temperature, which is furthermore influenced by oligomers in the polymer matrix.^51^

The first weight loss of the SPE starts at about 305 °C, which is due to the structural decomposition of the acrylate matrix. The I-SPEs show an initial decomposition at lower temperatures (around 260 °C), which continues up to about 390 °C. It is most likely that the structural decomposition starts at said temperature point. The final weight loss of the membranes at approximately 390–470 °C is due to the decomposition of LiTFSI. In relation to the SPE, the thermal stability of the I-SPEs decreases after electropolymerization.

The glass transition temperatures of the second heating cycle are depicted in Fig. 5b. The materials show similar behavior in terms of thermal transition. The *T*_g_-analysis provides values around −20 °C. Apart from the glass transition, there are no explicit thermic effects present in the temperature range from −50 to 150 °C. The lack of a melting peak concludes that the polymer matrix, as well as the composite materials, exhibit amorphous behaviour. It should be pointed out that the first heating cycles of all samples show a broad endothermic peak, which could be interpreted as melting peaks. Nevertheless, there are no crystallization peaks in both cooling cycles. This is most likely due to the semi-crystallinity of the polymer after the preparation *via* UV-irradiation. Re-heating promotes the reorganization of morphologies in the material and leads to a decrease in crystalline regions. Regarding the thermal history of the polymer, only the important results from the second heating cycle were examined.^52^

### 3.3 Morphological properties

**Fig. 6:**
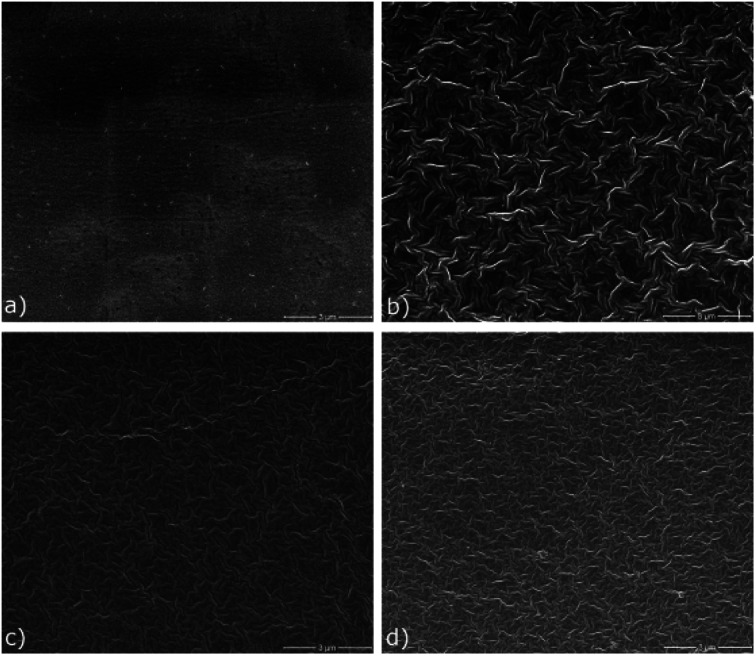
Top-view SEM images of (a) SPE, (b) I-SPE-oPPy, (c) I-SPE-oPMePy and (d) I-SPE-oPEDOT at identical settings.

Fig. 6 shows the SEM images of the SPE and the I-SPEs at identical settings. Significant differences can be observed after the electropolymerization procedure between the polymer matrix and the interpenetrated network. There are distinct morphological differences visible on the surface of the materials. The polymer matrix SPE (a) shows a very dense surface that incorporates a few inhomogen regions with pores (areas with brighter contrast). Many small particles, presumably lithium salt crystals, are present on the surface of the polymer. This indicates a poor dispersion of the LiTFSI within the acrylate matrix. On the other hand, the polymer composites show a rather uniform surface, but with much thicker polymer chains. They form a well-defined 3D-framework with noticeably larger gaps between each polymer fragment. The I-SPE-oPEDOT membrane (d) exhibits also some crystals, which are attached to the polymer chains. A relation between the dissociation of the lithium salt in the polymer matrix and the ionic conductivity is dominant, since the dissolution leads to a higher number of free ions for the lithium transport.^53^ The pyrrole-derivates I-SPE-oPPy (b) and I-SPE-oPMePy (c) present a uniform network without any depositions. Hence, a correlation between the structural analysis and the ionic conductivity of the materials is noticeable.

### 3.4 Electrochemical properties

**Fig. 7:**
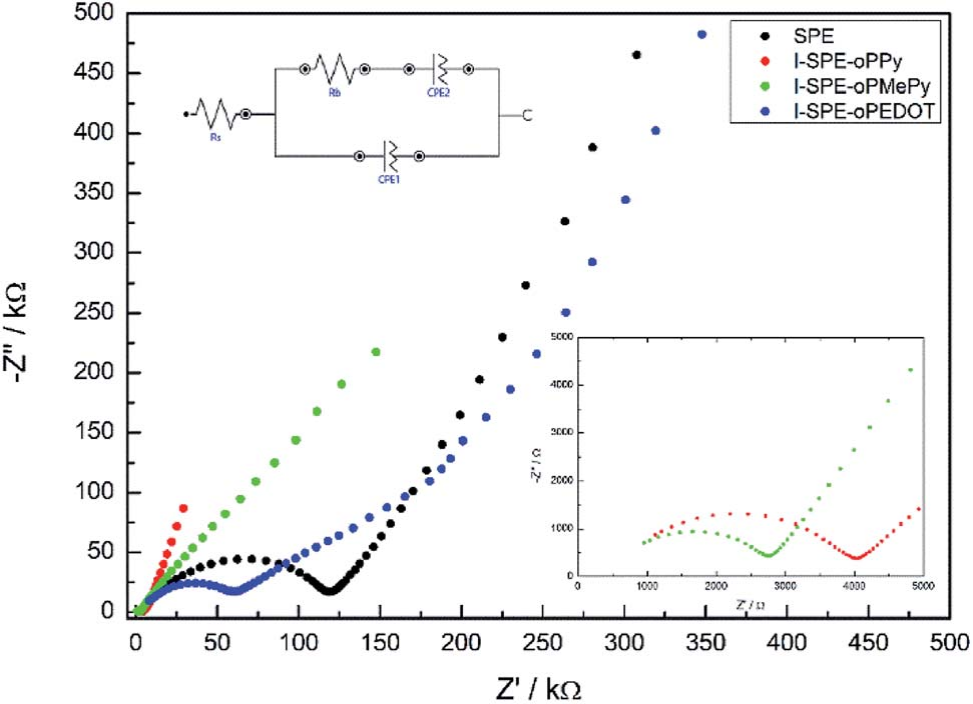
Nyquist plots of the prepared samples at RT.

**Fig. 8:**
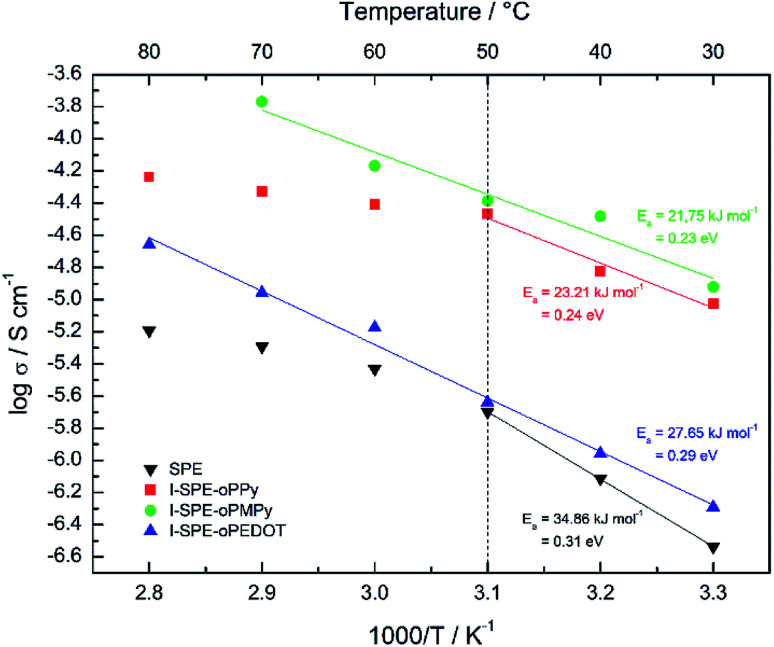
Arrhenius plots for the SPE, I-SPE-oPPy, I-SPE-oPMePy and I-SPE-oPEDOT.

The ratio of the main components of the SPE is crucial and affects the properties drastically, *e.g.* the ionic conductivity and the mechanical-, thermal- and electrochemical stability. Different compositions of the components were tested to enhance the ionic conductivity of the polymer matrix. The LiTFSI content was kept constant at 10 wt%. A GPTA : PEGDA ratio of 1 : 4 was found to have the highest ionic conductivity for the SPE matrix. The total amount of heterocyclic monomer (Py, MePy or EDOT) added to the formulation was fixed at 20 wt%, because of the notably increasing brittleness of the polymer film with higher monomer content. The ionic conductivity of the SPE reached a value of 1.3 × 10^−7^ S cm^−1^ at RT. The preparation of the I-SPE, incorporating overoxidized PPy, PMePy or PEDOT within the acrylate matrix, leads to an enhanced ionic conductivity up to 6.1 × 10^−6^ S cm^−1^ at RT in the case of I-SPE-oPMePy. The ionic conductivity of the I-SPE-oPPy and the I-SPE-oPEDOT is 3.5 × 10^−6^ S cm^−1^ and 1.8 × 10^−7^ S cm^−1^, respectively. Fig. 7 shows the Nyquist diagram of the different composites at RT. The equivalent circuit (inset of Fig. 7) was simplified as much as possible to describe the actual polymeric system, where *R*_S_ is the solution resistance, *R*_b_ the bulk resistance, CPE_1_, and CPE_2_ the constant phase elements. It can be assumed, that the rise in ionic conductivity is influenced by the dissociation of the lithium salt in the polymer matrix by the addition of Py, MePy or EDOT as disperse agent. After the polymerization, the interaction of the TFSI^−^ anion with the heteroatom of the polymer backbone leads to an increase in available charge carriers.^54^ The temperature-dependent ionic conductivities of the different materials are shown in Fig. 8. The Arrhenius plot depicts the increasing conductivity with rising temperature from 30 to 80 °C, which follows the trend of the room temperature conductivity measurements. At 70 °C, I-SPE-oPMePy exhibits a high ionic conductivity of 7 × 10^−4^ S cm^−1^, whereas the ionic conductivity of the SPE is about two orders of magnitude lower (5.1 × 10^−6^ S cm^−1^). In general, PEO-based solid polymer electrolytes are known for their cation transport through segmental motion of PEO chains, which depends on the amount of amorphous regions. An increase in amorphous regions with raised temperature leads to higher ionic conductivity. The curvature in the plots of SPE and I-SPE-oPPy reveals an inflection point around 50 °C. I-SPE-oPMePy and I-SPE-oPEDOT show a linear increase in conductivity with rising temperature. This difference could be related with increased amorphousity of these two IPNs. Furthermore, the linear course of the plots (low temperature behaviour of SPE and I-SPE-oPPy) corresponds to the Arrhenius equation. The calculated activation energy from the linear fittings indicates that the lithium ion transfer is favored with increasing ionic conductivity, because of the lower energy barrier. Thus, I-SPE-oPMePy exhibits the lowest activation energy for lithium ion conduction (0.23 eV), followed by I-SPE-oPPy (0.24 eV), I-SPE-oPEDOT (0.29 eV) and SPE (0.31 eV).

## 4. Conclusions

In this work, we report the preparation of an interpenetrating polymer network *via* combined *in situ* UV- and electropolymerization. The conductive polymers poly(1*H*-pyrrole), poly(*N*-methylpyrrole) and poly(3,4-ethylenedioxythiophene) were encapsuled in a solid polymer matrix and overoxidized subsequently by an electrochemical procedure. The aim was to create a basic 3D polymer matrix for the proposed *in situ* electropolymerization as a proof of concept material. The experimental results show that the electron-rich groups are introduced onto conducting polymer units during overoxidation treatment and that the conducting polymer was converted into an ion-exchange membrane. The I-SPEs exhibited better ionic conductivity (up to 6.1 × 10^−6^ S cm^−1^ for I-SPE-oPMePy) at room temperature compared to the pure SPE (1.3 × 10^−7^ S cm^−1^). Temperature-dependent conductivity measurements showed a high ionic conductivity of 7 × 10^−4^ S cm^−1^ at 70 °C for I-SPE-oPMePy. The calculated activation energies for lithium ion conduction display the effect of the generated IPNs, which lower the energy barrier for Li^+^ conduction. The activation energy was lowered by 0.08 eV in the case of I-SPE-oPMePy, compared to the SPE. The permeability of the overoxidized conductive polymer can be controlled by changing the overoxidation conditions. The overoxidation of the I-SPEs was identified by characteristic peaks in the ATR-FT-IR spectra. Beside the major effect on the ionic conductivity, no critical changes of the SPE properties were observed, which is important, as the conductivity is the adjusting parameter. Such materials could lead to a novel group of interpenetrated solid polymer electrolytes as separator materials based on mixed conductors. Moreover, the combination of a polyacrylate-matrix with cation selective properties of overoxidized conducting polymers leads to 3D materials with higher ionic conductivity than SPEs and selective ion-exchange membrane properties with good stability by facile fabrication. These kinds of synthesis methods appear to provide an alternative way of varying the properties and applications of conducting polymers.

## Conflicts of interest

There are no conflicts to declare.

## Supplementary Material

RA-010-D0RA07966A-s001
