# Supplementary material for: Ionic conductivity enhancement in solid polymer electrolytes by electrochemical in situ formation of an interpenetrating network
Source: RSC Adv. 2020 Nov 12;10(68):41296–304. doi: 10.1039/d0ra07966a (PMC9057755; doi:10.1039/d0ra07966a)
Supplement: RA-010-D0RA07966A-s001 [file RA-010-D0RA07966A-s001.pdf]

Electronic Supplementary Material (ESI) for RSC Advances.
This journal is © The Royal Society of Chemistry 2020

Electronic Supplementary Information available

"Ionic conductivity enhancement in solid polymer electrolytes by electrochemical *in situ* formation of an interpenetrating network"

by Kristian Leš and Carmen-Simona Jordan*

(* = corresponding author)
By

University of Applied Science Osnabrück, Albrechtstr. 30, 49076 Osnabrück, Germany.
E-mail: k.les@hs-osnabrueck.de, s.jordan@hs-osnabrueck.de

Correspondence to:

* Prof. Dr. habil. Carmen-Simona Jordan,

University of Applied Science Osnabrück, Albrechtstr. 30, 49076 Osnabrück, Germany.
Phone: +49-591-80098-296, s.jordan@hs-osnabrueck.de, www.ids.hs-osnabrueck.de

Table of Contents

S1. Impedance profiles of a) SPE, b) I-SPE-oPPy, c) I-SPE-oPMePy and d) I-SPE-oPEDOT at different temperatures.

S2. Arrhenius plot of a) SPE and b) I-SPE-oPPy before (non-humid) and after storage in a humid environment for 24 hours and impedance profiles of c) SPE (humid) and d) I-SPE-oPPy (humid).

S3. Electrochemical synthesis and overoxidation of PPy on glassy carbon electrode as example.

S4. a) Oxidation and overoxidation of PPy and b) determination of the degree of overoxidation.

S5. Study of the electroactivity of modified oPPy/GC electrode by cyclic voltammetry, using a) the negatively charged redox marker $[Fe(CN)_6]^{4-}$ and b) the positively charged redox marker $[Ru(NH_3)_6]^{3+}$ in relation to c) the non-overoxidized PPy.

S6. References

S1. Impedance profiles of a) SPE, b) I-SPE-oPPy, c) I-SPE-oPMePy and d) I-SPE-oPEDOT at different temperatures.

The electrochemical impedance spectroscopy measurements were performed within a frequency range of 10^6 Hz to 1 Hz with an AC amplitude of 20 mV. Equivalent circuit for SPE: [R(RQ)Q]. Equivalent circuit for I-SPEs: [R(RQ)(RQ)Q], with *R* = resistance and *Q* = constant phase element.

a)

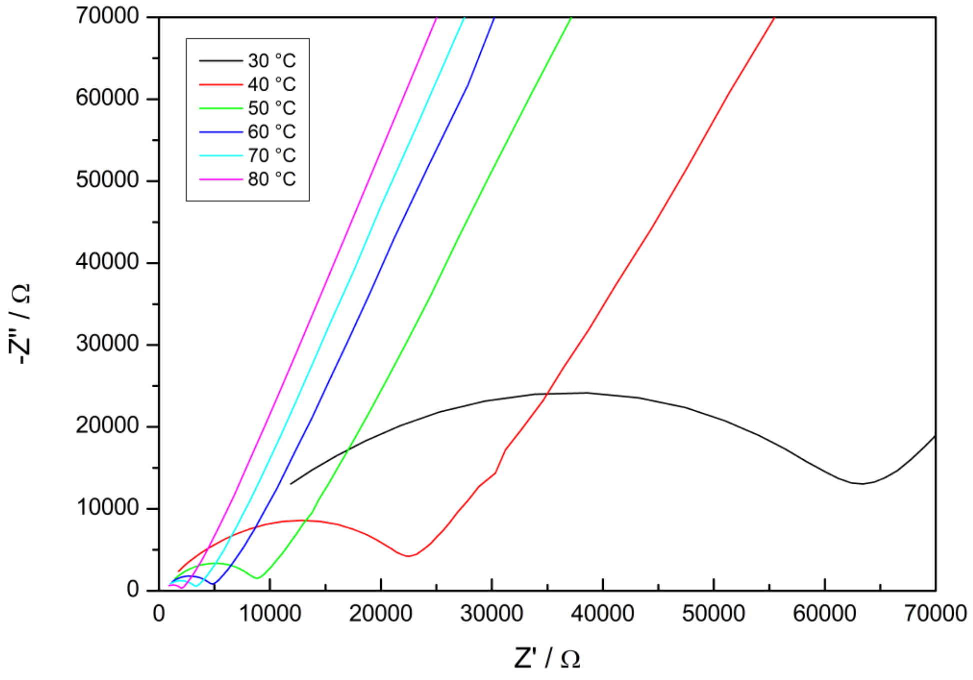

b)

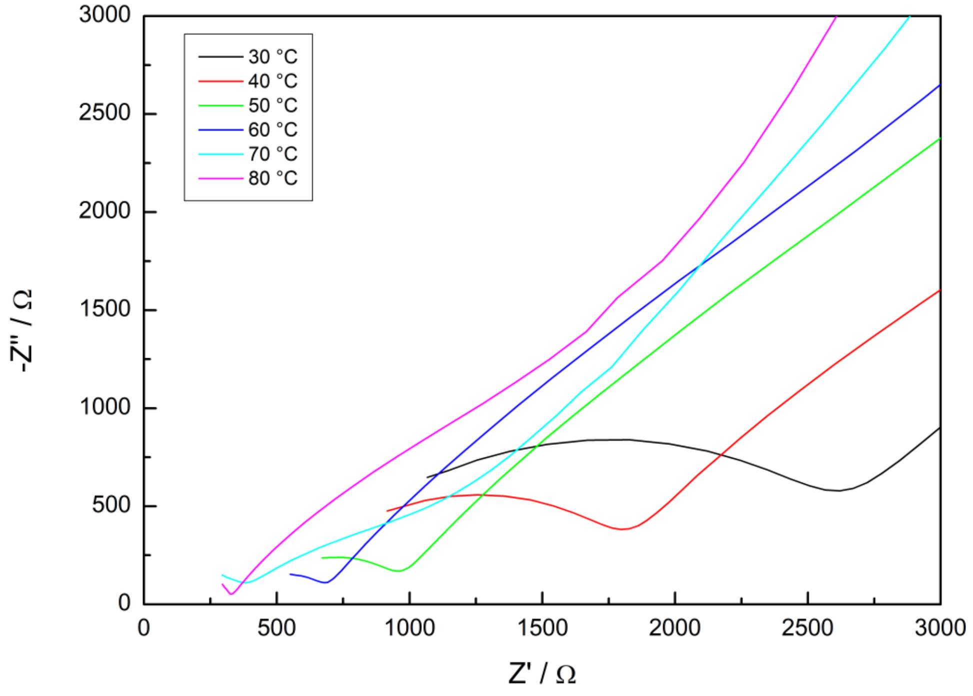

c)

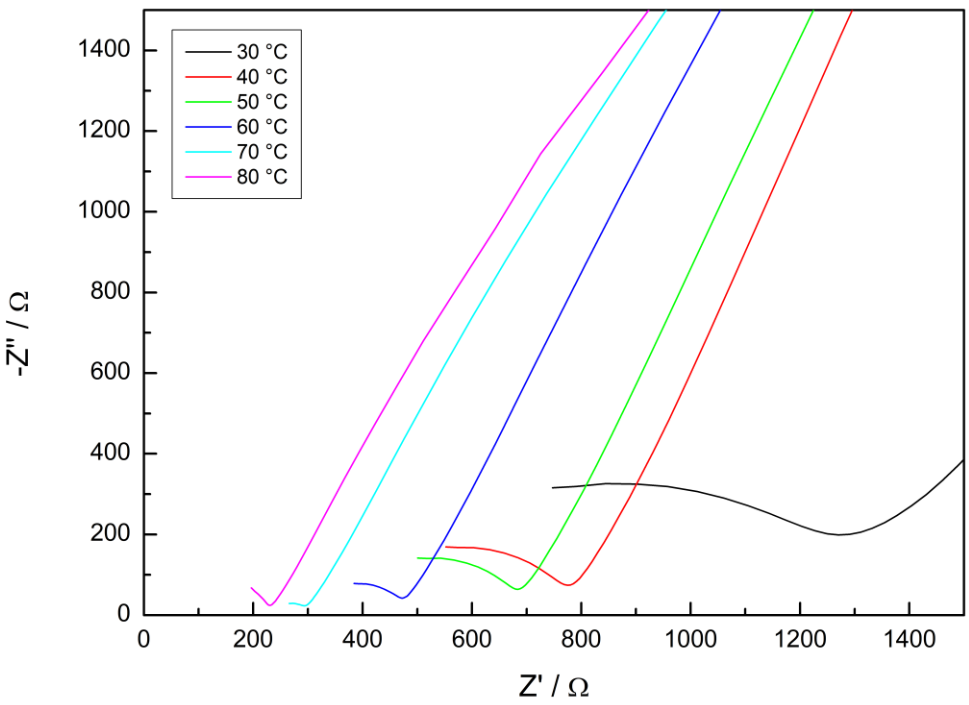
30 °C
40 °C
50 °C
60 °C
70 °C
80 °C
-Z'' / Ω
Z' / Ω
0
200
400
600
800
1000
1200
1400

d)

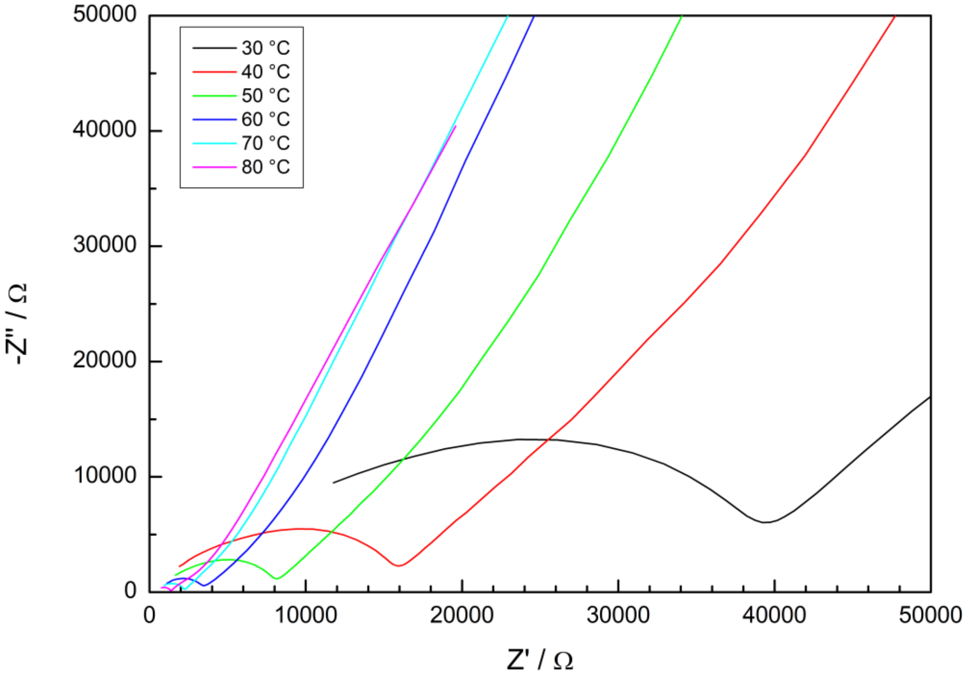
30 °C
40 °C
50 °C
60 °C
70 °C
80 °C
-Z'' / Ω
Z' / Ω
0
10000
20000
30000
40000
50000

S2. Arrhenius plot of a) SPE and b) I-SPE-oPPy before (non-humid) and after storage in a humid environment for 24 hours and impedance profiles of c) SPE (humid) and d) I-SPE-oPPy (humid).

The electrochemical impedance spectroscopy measurements were performed within a frequency range of 10^6 Hz to 1 Hz with an AC amplitude of 20 mV. Equivalent circuit for SPE: [R(RQ)Q] at low temperature and [LR(QR)] at higher temperatures. Equivalent circuit for I-SPE-oPPy: [R(RQ)Q], with *R* = resistor, *Q* = constant phase element and *L* = inductor.

a)

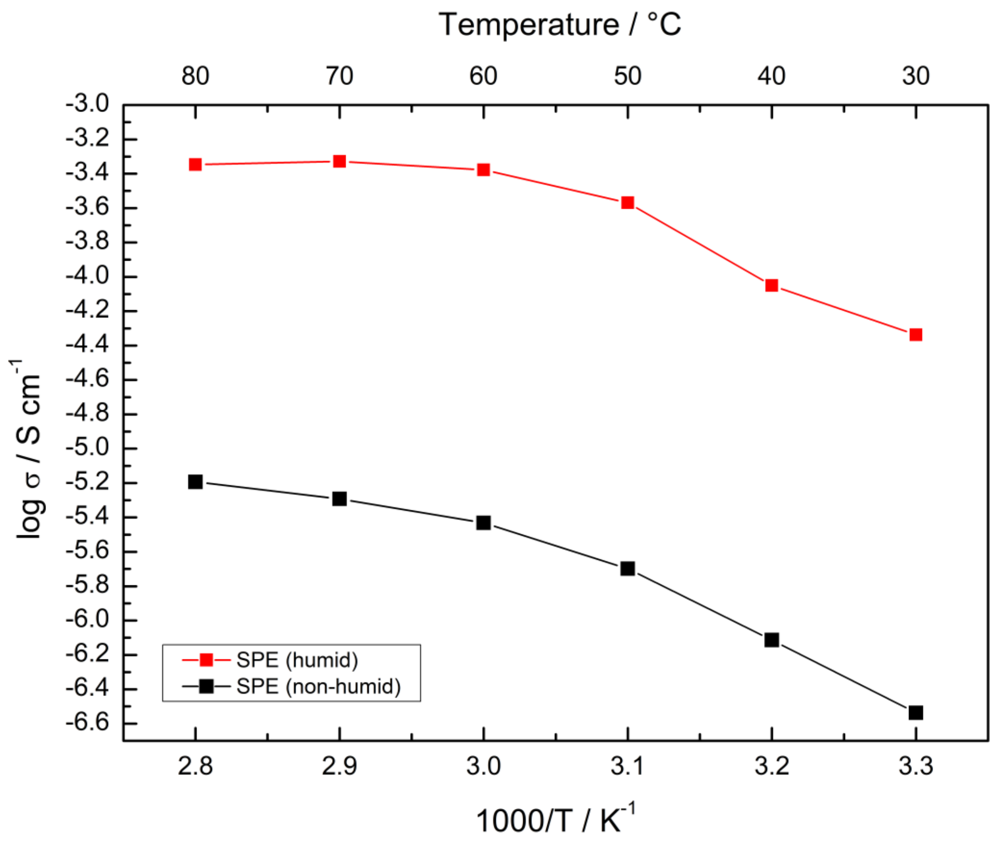

b)

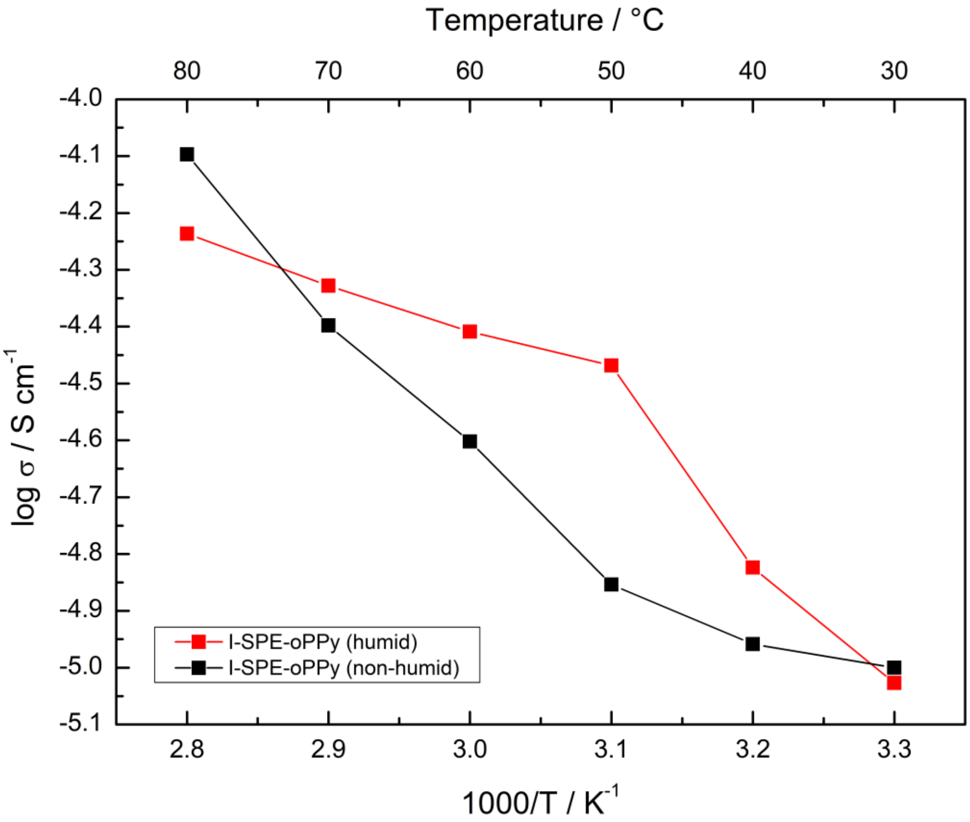

Temperature / °C
80
70
60
50
40
30
-4.0
-4.1
-4.2
-4.3
-4.4
-4.5
-4.6
-4.7
-4.8
-4.9
-5.0
-5.1
log σ / S cm-1
I-SPE-oPPy (humid)
I-SPE-oPPy (non-humid)
2.8
2.9
3.0
3.1
3.2
3.3
1000/T / K-1

c)

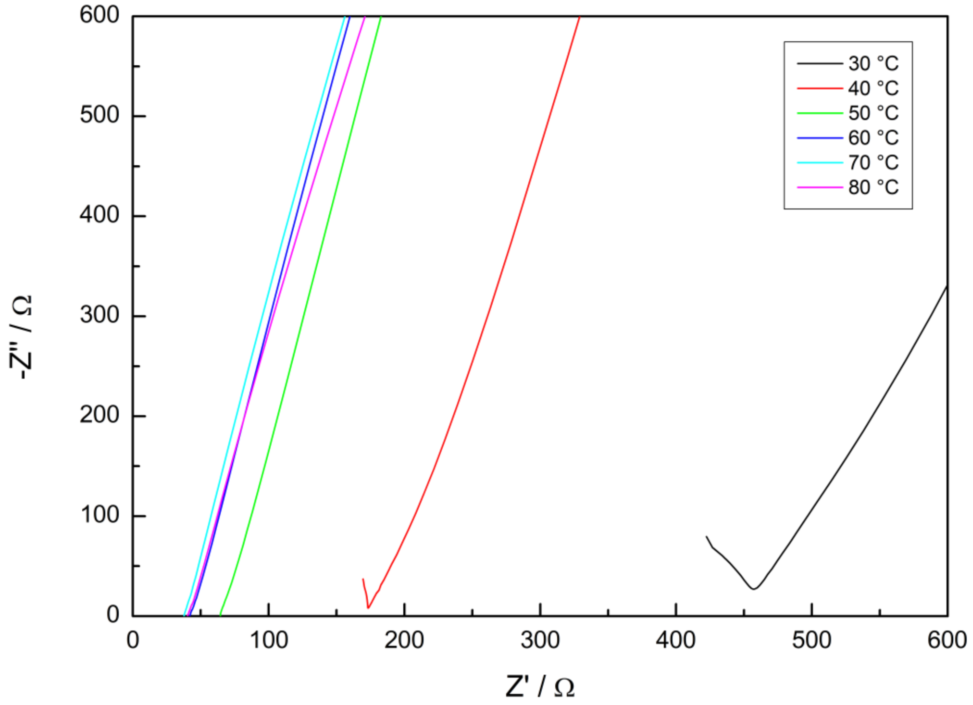

600
500
400
300
200
100
0
-Z'' / Ω
30 °C
40 °C
50 °C
60 °C
70 °C
80 °C
0
100
200
300
400
500
600
Z' / Ω

d)

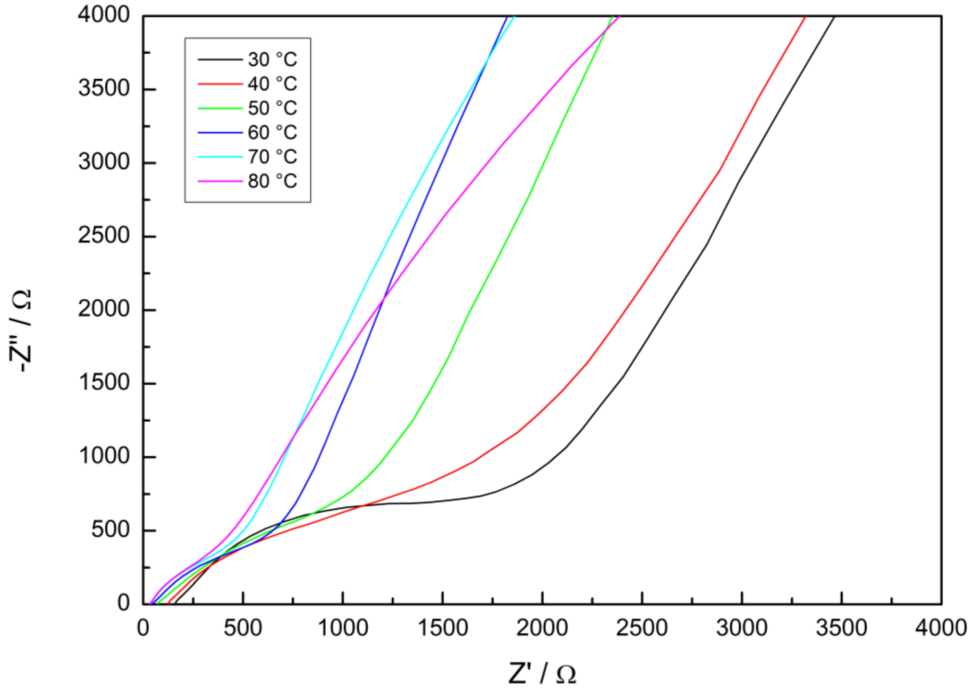

S3. Electrochemical synthesis and overoxidation of PPy on glassy carbon electrode as example.

The electrodeposition was performed in a three-electrode setup with glassy carbon as working electrode, a platinum-sheet counter electrode and Ag/AgCl as reference electrode. The electrolyte was composed of 0.2 M LiTFSI and 0.1 M pyrrole in acetonitrile. The measurement was performed with a scan rate of 100 mV/s between -0.5 and 1.2 V for 15 cycles. The overoxidation was performed in an electrolyte composed of 0.2 M LiTFSI in acetonitrile at a constant potential of 2.2 V for 2000 s.

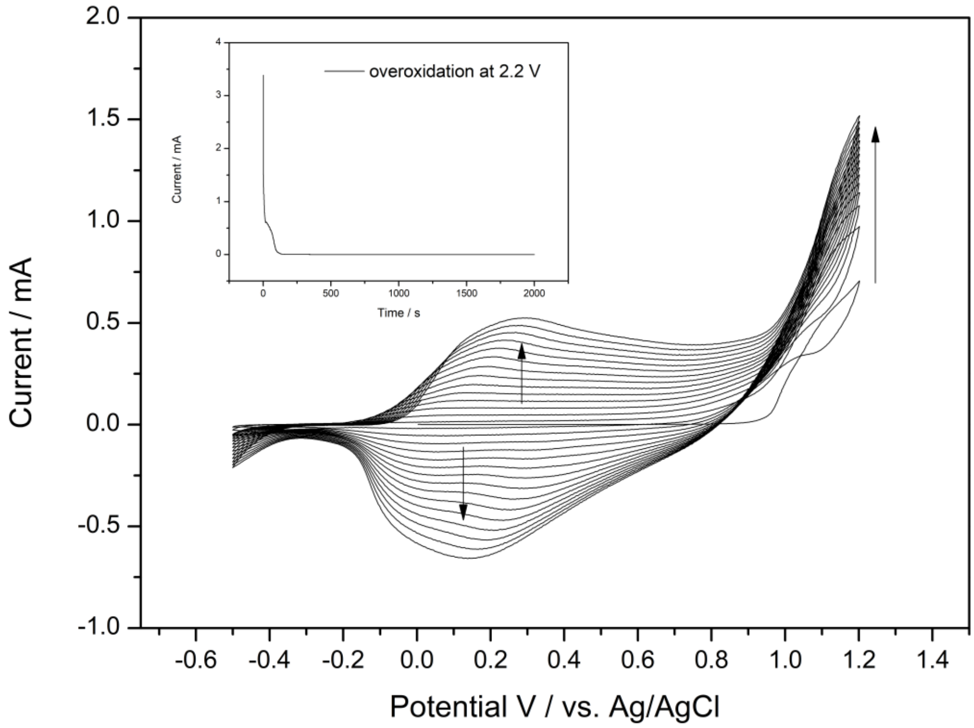

S4. a) Oxidation and overoxidation of PPy and b) determination of the degree of overoxidation.

The degree of oxidation of the deposited PPy on the GC electrode was examined in a monomer-free electrolyte solution of 0.2 M LiTFSI in acetonitrile at different potentials with a platinum-sheet counter electrode and a Ag/AgCl reference electrode. The charge ratio (Q_-/Q_+) at each potential was calculated by division of the integral areas of positive and negative charge. A value of 1 indicates oxidation, whereas a value smaller than 1 indicates increasing overoxidation.[1]

a)

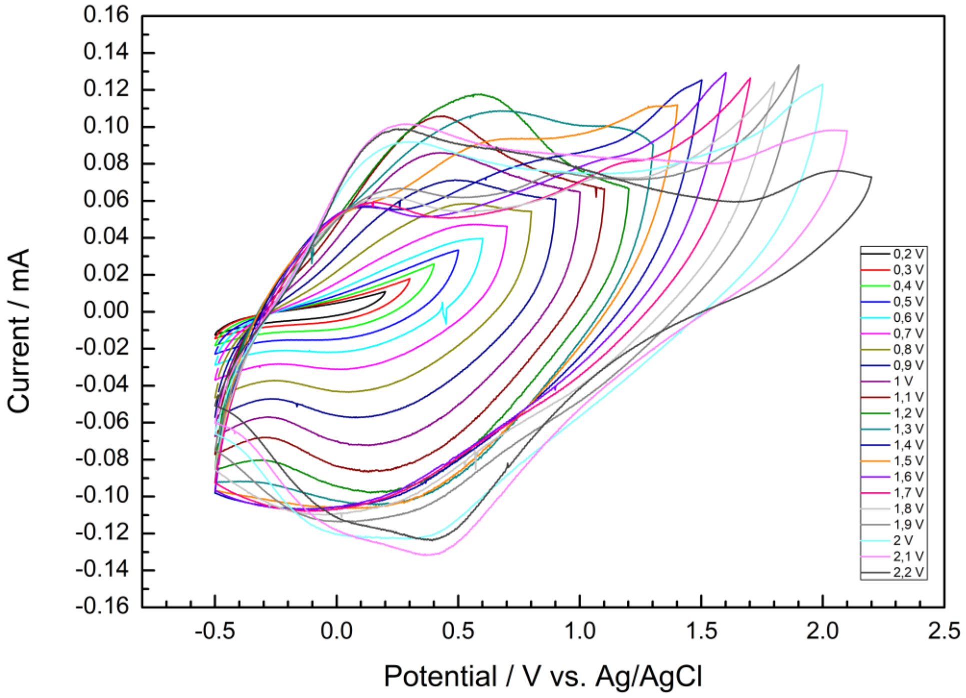

b)

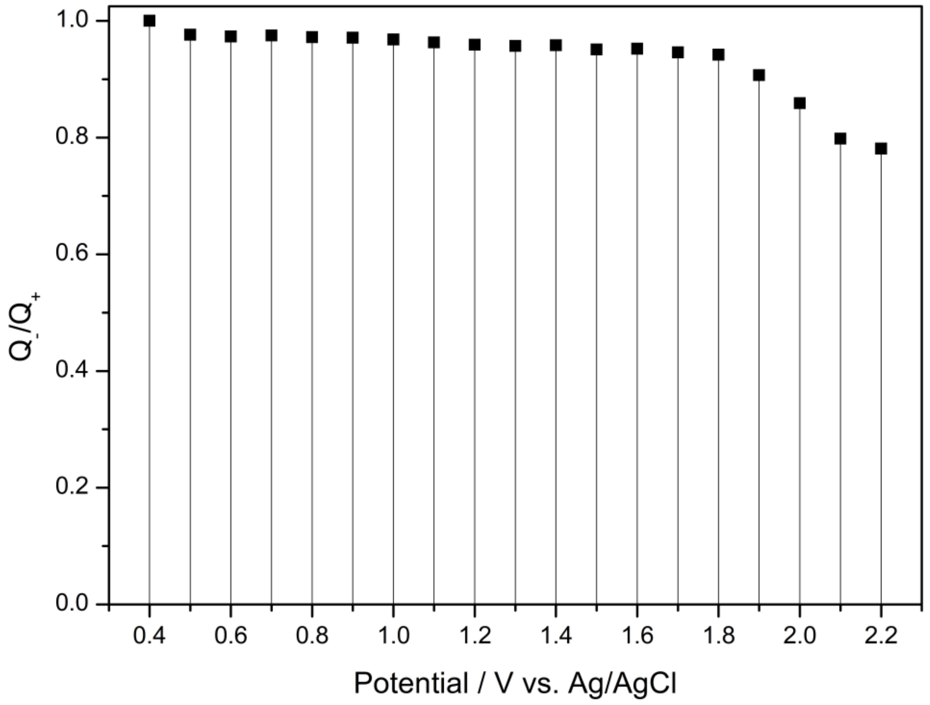

S5. Study of the electroactivity of modified oPPy/GC electrode by cyclic voltammetry, using a) the negatively charged redox marker $[Fe(CN)_6]^{4-}$ and b) the positively charged redox marker $[Ru(NH_3)_6]^{3+}$ in relation to c) the non-overoxidized PPy.

The electroactivity of overoxidized PPy on a glassy carbon electrode was examined.[2] First, the oPPy was deposited on glassy carbon according to the procedure from S3. The oPPy/GC electrode was then immersed and examined in different aqueous electrolyte solutions composed of a) 1 mM $K_4[Fe(CN)_6]$ and 0.1 M KCl and b) 1 mM $Cl_3[Ru(NH_3)_6]$ and 0.1 M KCl. A cyclic voltammogram was recorded (platinum-sheet counter electrode and Ag/AgCl as reference electrode) at a scan rate of 10 mV/s between -0.1 and 0.5 V. In relation to the overoxidized PPy, a cyclic voltammogram was recorded between -0.5 and 0.5 V at a scan rate of 50 mV/s with non-overoxidized PPy (c).

a)

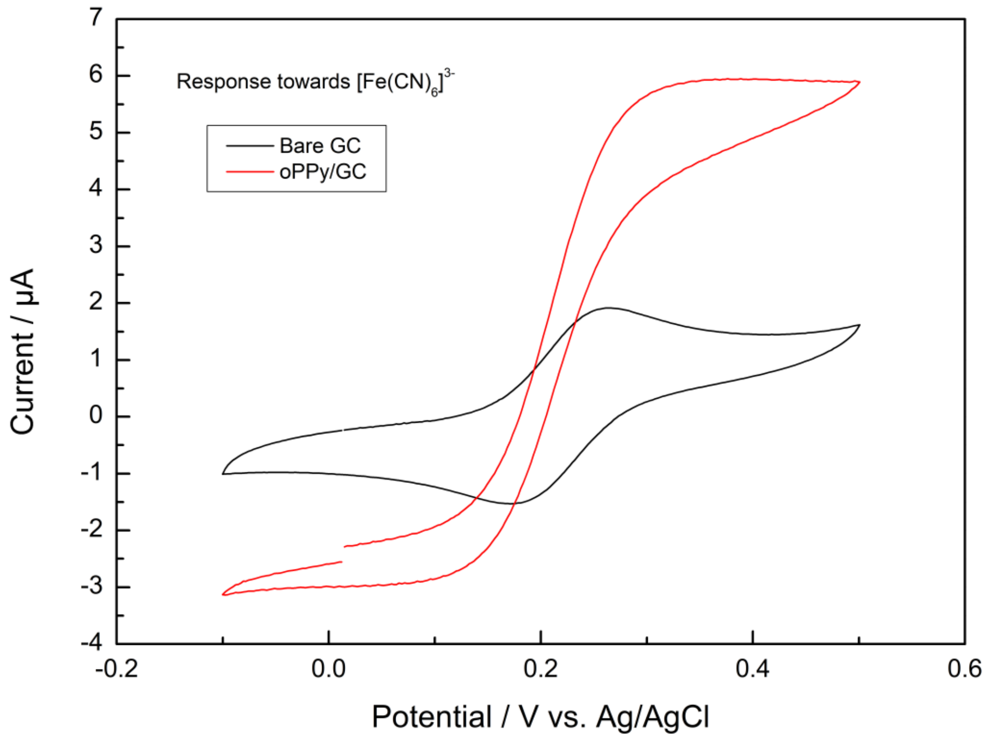

b)

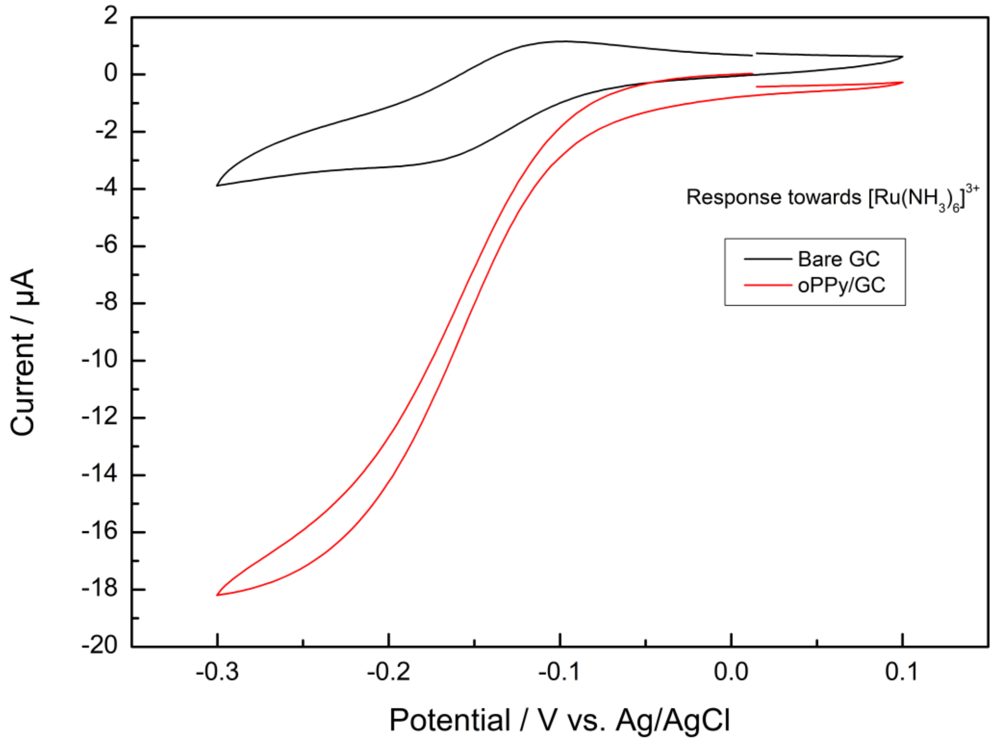
Response towards $[Ru(NH_3)_6]^{3+}$
Bare GC
oPPy/GC
Current / µA
Potential / V vs. Ag/AgCl
2
0
-2
-4
-6
-8
-10
-12
-14
-16
-18
-20
-0.3
-0.2
-0.1
0.0
0.1

c)

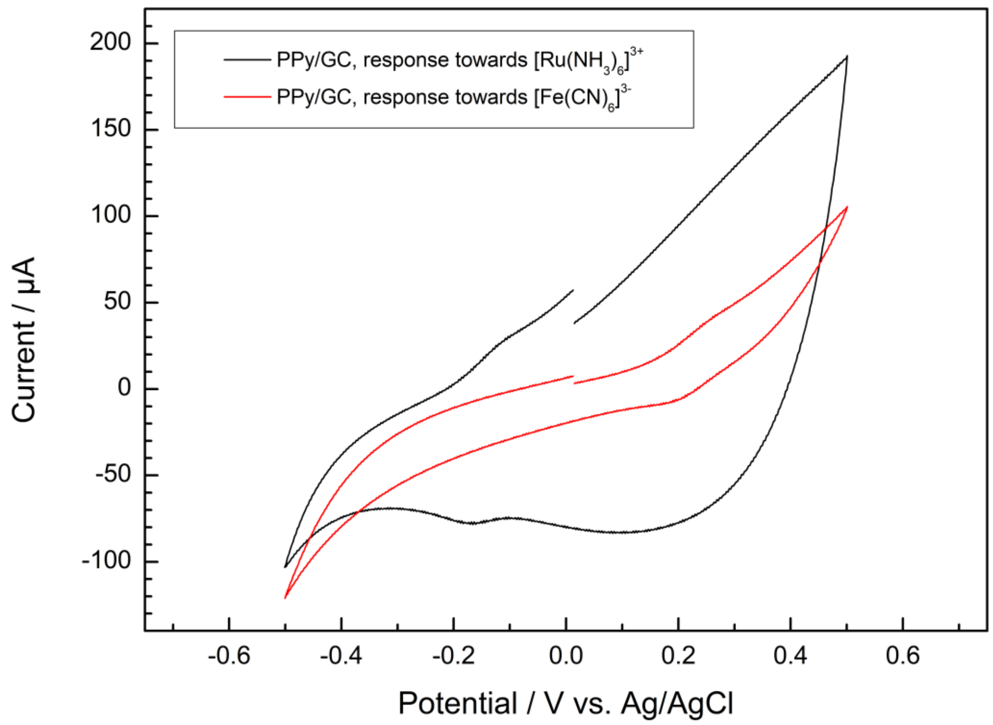
PPy/GC, response towards $[Ru(NH_3)_6]^{3+}$
PPy/GC, response towards $[Fe(CN)_6]^{3-}$
Current / µA
Potential / V vs. Ag/AgCl
200
150
100
50
0
-50
-100
-0.6
-0.4
-0.2
0.0
0.2
0.4
0.6

S6. References

1 M. Skompska, M. A. Vorotyntsev, M. Refczynska, J. Goux, E. Lesniewska, G. Boni, C. Moise, *Electrochim. Acta*., 2006, **51**, 2108-2119.

2 S. Mondal and M. V. Sangaranarayanan, *Phys. Chem. Chem. Phys*., 2016, **18**, 30705-30720.
